# Effects of dietary fibers varying in physicochemical properties on total endogenous protein losses and protein digestibility in broilers

**DOI:** 10.1016/j.psj.2026.106902

**Published:** 2026-04-07

**Authors:** Mochammad F. Habibi, Sebastian Dorado-Montenegro, Ana Isabel García-Ruiz, Walter J.J. Gerrits, Despoina Georgaki, René P. Kwakkel, Sonja de Vries

**Affiliations:** a Animal Nutrition Group, Wageningen University & Research, P.O. Box 338, 6700 AH, Wageningen, the Netherlands; b Faculty of Animal Science, Universitas Gadjah Mada, Yogyakarta, 55281, Indonesia; c Escuela de Zootecnia, Universidad de Costa Rica, San José, Costa Rica, 2060, San José, Costa Rica; d Poultry R&D, Trouw Nutrition, El Viso de San Juan, Toledo, 45215, Spain

**Keywords:** Chicken, Non-starch polysaccharide, Viscosity, Digestion processes, Nutrient digestion

## Abstract

This study aimed to quantify endogenous protein losses (**EPL**) and protein digestibility in broilers fed diets containing dietary fibers (**DF**) differing in particle size (**PS**) and solubility. A total of 360 female 1-d-old Ross 308 broilers were randomly allocated to 40 pens (9 birds/pen). From d 10, broilers received one of five dietary treatments. Four diets were tested in a 2 × 2 factorial design containing 120 g/kg coarse or fine soybean hulls (**SBH**) as an insoluble DF source, with or without 50 g/kg purified wheat arabinoxylans (**AX**) as a viscous soluble DF source. A fifth diet replaced fine SBH with 150 g/kg fine sugar beet pulp (**SBP**), a DF source with notable hydration properties. Between d 26 and 31, three birds per pen received a daily oral dose of a ^15^N-isotope solution containing 1.2 g ^15^N-enriched milk protein concentrate (2.27 atom %) and 0.12 g ^15^NH_4_Cl (≥98 atom %), with an initial dose of milk protein concentrate of 2.4 g at d 26. Six days after ^15^N-solution withdrawal (d 37), excreta were collected, birds were euthanized, and ileal digesta were sampled. Arabinoxylans increased EPL in feces (+0.35 g/kg DM intake, *P* = 0.020) and reduced true ileal protein digestibility (−2.45%-units, *P* < 0.001), regardless of SBH PS. Apparent ileal protein digestibility decreased only when AX was added to the fine SBH diet (PS × AX interaction, *P* = 0.024). Replacing fine SBH with fine SBP increased EPL in ileal digesta (+0.31 g/kg DM intake, *P* = 0.002) and feces (+0.92 g/kg DM intake, *P* = 0.004), without affecting true protein digestibility. Overall, both AX and SBP increased EPL in broilers, but only AX reduced true protein digestibility. The PS × AX interaction suggests that the viscous soluble DF impact depends on PS of the dietary matrix. These results highlight the importance of considering the counteracting DF effects on EPL and nutrient absorption, as apparent digestibility alone is insufficient to capture these responses.

## Introduction

The use of fiber-rich by-products from agricultural and food industries has gained attention in the poultry feed sector as a strategy to enhance food security, sustainability, and animal welfare. Dietary fibers (**DF**) have been shown to modulate endogenous protein losses (**EPL**) and digestibility of nutrients in chickens (reviewed by Jha and Mishra, 2021) due to their physicochemical properties (reviewed by Eggum, 1995) and understanding these effects is essential when considering inclusion of DF-rich ingredients into poultry diets. Inherent to the structural diversity of DF, the effects of DF-rich ingredients on EPL and nutrient digestion are, however, highly variable.

For example, the structural characteristics of insoluble DF (**iDF**) sources, including hardness - or resistance to breakdown, have been known to influence digesta transit behavior and nutrient digestibility (Ferrando et al., 1987; González-Alvarado et al., 2007; Jiménez-Moreno et al., 2009; Mateos et al., 2012; Akbari Moghaddam Kakhki et al., 2024). Particles that resist physical and chemical degradation in the upper gastrointestinal tract (**GIT**) stimulate gizzard contraction, promoting muscular development, prolonging digesta retention time, and enhancing enzyme–substrate interactions, which may ultimately improve nutrient digestibility (Hetland and Svihus, 2001; González-Alvarado et al., 2007; Jiménez-Moreno et al., 2009; Svihus, 2011). The modification of particle size (**PS**) of iDF sources is a practical strategy to exploit these structural effects (Mosenthin et al., 1994; Lahaye et al., 2008; reviewed by Jha and Mishra, 2021). For instance, coarse oat hulls, which provide larger and harder particles, have been shown to increase gizzard weight, prolong digesta retention time, and finally may improve nutrient digestibility when compared with finely ground oat hulls (Hetland and Svihus, 2001; Svihus, 2011; Naeem et al., 2023). Nevertheless, whereas enhanced gizzard function may facilitate nutrient digestion, as reflected by increased true protein digestibility, iDF may also elevate EPL due to the increased secretion of digestive enzymes (Svihus, 2011; Yokhana et al., 2016), increased thickness of the gastrointestinal mucus layer, and the abrasive action of coarse particles on the epithelial mucosa (Leterme et al., 1998b; Oryschak et al., 2002).

Soluble DF (**sDF**) and DF source with noticeable hydration properties, including high water-binding capacity (**WBC**), may also increase EPL and decrease nutrient digestibility, although the mode of action may differ from iDF (Souffrant, 2001; Dégen et al., 2007). Soluble DF typically increase digesta viscosity, which may enhance cell proliferation in the small intestine (Gee et al., 1996; Wanders et al., 2013, 2014), hinder the reabsorption of bile acids and digestive enzymes in the ileum (Souffrant, 2001; Adeola et al., 2016; reviewed by Jha and Mishra, 2021), resulting in increased EPL. Furthermore, computer simulations revealed that high digesta viscosity may lower nutrient diffusion and hamper intestinal contractions along the small intestine (Henn and Alim, 2025), hence potentially reducing nutrient hydrolysis, limiting transport through the gut lumen, decreasing interactions with the epithelial surface, and ultimately impairing nutrient absorption.

Our current understanding of DF-induced effects on nutrient digestibility is largely based on apparent digestibility estimates (Mateos et al., 2012; Vivares et al., 2025), which are routinely evaluated to assess the nutritional quality of feed ingredients. By influencing EPL and true digestibility of protein, however, DF may affect apparent digestibility values in various ways, depending on their specific physicochemical properties such as PS, solubility, and gel forming- and hydration characteristics. Because apparent protein digestibility values reflect the combined effects of EPL and true protein digestibility, they provide no insights into the underlying mode of action and - when EPL and true digestibility change in opposite directions - may even mask the effects of DF.

To better interpret effects of DF on apparent protein digestibility reported in literature, we need to obtain insights in the diverse effects of DF on (1) EPL vs. (2) hydrolysis and absorption of protein, i.e. true digestibility. To address this, the objective of this study was to investigate how DF sources varying in physicochemical properties influence EPL and true digestibility of protein in broilers. As a model for cereal-derived soluble DF, we used isolated arabinoxylans from wheat, as soluble polysaccharides with strong gelling properties (Mendez-Encinas et al., 2018), to induce variation in digesta viscosity and passage behavior, anticipating effects on EPL and protein digestion. Arabinoxylans were added to maize-based diets containing either coarse or fine soybean hulls as the main iDF source, enabling evaluation of the effects of supplementing viscous sDF to iDF differing in PS. Soybean hulls contain ∼64% of non-starch polysaccharides (**NSP**), out of which ∼70% are water-insoluble and ∼30% are water-soluble, but compared with other iDF sources, such as oat hulls, soy bean hulls may be more fermentable (Hsu et al., 1987; Lo et al., 1989; Akbari Moghaddam Kakhki et al., 2024). It was expected that viscous sDF in the presence of fine soybean hulls would differently modulate digesta properties than in the presence of coarse SBH, as the viscosity of suspensions is very sensitive to the size of particles present (Senapati et al., 2010; Konijn et al., 2014). A fifth diet was added where ground soybean hulls were replaced with ground sugar beet pulp, a DF source containing a ∼61% NSP composed of heterogeneous water-insoluble (∼60% of NSP) and soluble (∼40% of NSP) polysaccharides linked within its plant cell wall matrix, with pectic polysaccharides, arabinans, and cellulose as the major constituents (Zykwinska et al., 2006; Akbari Moghaddam Kakhki et al., 2024). Sugar beet pulp has low lignin-content, exerting noticeable hydration properties but low gelling properties and viscosity (Phatak et al., 1988). Hence, this diet allowed evaluation of the effects of an insoluble DF source with markedly different characteristics from cereal-derived arabinoxylans.

## Materials and methods

The experiment was performed at the research facility Carus, Wageningen University & Research, Wageningen, The Netherlands in June 2021. Experimental procedures were approved by the Dutch Central Committee of Animal Experiments, the Netherlands, under the authorization number AVD1040020197324 and in accordance with the Dutch Act on Animal Experimentation and EU Directive 2010/63/EU. This study was part of a larger animal experiment on the use of minimally-invasive ^15^N-isotope dilution method and the effects of DF on digesta retention time and apparent nutrient digestibility (Dorado-Montenegro et al., 2024; Habibi et al., 2026).

### Birds and housing

A total of 360 female (Ross 308) one-d-old broilers (initial BW: 40.7 ± 3.22 g) were obtained from a commercial hatchery. Each bird was individually weighed, wing-tagged, and randomly allocated to one of 40 pens (9 birds/pen). The pens were then assigned to one of five dietary treatments, resulting in 8 replicate pens per treatment. Pens (1.1 m × 1.85 m) were equipped with plastic floating slatted floors covered with rubber matting, bedded with wood shavings, and equipped with a perch, feeder, and five drinking nipples connected to a 5-L water tank. At d 21, rubber matting and bedding were removed to prevent ingestion of bedding and plastic sheets were placed underneath the barn to collect the excreta for digesta retention time, apparent nutrient digestibility, EPL, and true protein digestibility evaluation. Birds had *ad libitum* access to feed and water. The room temperature was set at 32 °C from d 1 to d 3, and thereafter gradually decreased to 22 °C in wk 4 and maintained until the end of the experiment. The health condition of the birds was assessed daily.

### Experimental design and dietary treatments

In the first 10 d, all birds were fed a commercial pelleted maize-soybean based starter diet. From d 10 to 37, the pelleted experimental diets were offered. The effects of PS of soybean hulls (coarse vs. fine) and the addition of arabinoxylans (0 vs. 50 g/kg) were tested using a 2 × 2 factorial arrangement of treatments. A fifth diet was used to compare iDF sources varying in physicochemical properties, including WBC, by substituting fine soybean hulls with fine sugar beet pulp.

Soybean hulls (120 g/kg) either in coarse (**C-SBH**: intact SBH;) or fine (**F-SBH**: ground using a hammer mill to pass a 1 mm screen using LHM 20/16, 1.5 kW, Condux International, Mankato, United States of America; 1500 rpm) form, purified wheat arabinoxylans (**AX**) (50 g/kg) (NAXus®, BioActor BV, Maastricht, The Netherlands), and fine sugar beet pulp (150 g/kg) (**F-SBP**: ground twice to pass 4 mm and 1 mm screen using LHM 20/16, 1.5 kW, Condux International, Mankato, United States of America; 1500 rpm) were used as main DF sources. For the fifth diet, 150 g/kg F-SBP was substituted for 120.1 g/kg fine SBH, 28.4 g/kg sugar, and 1.5 g/kg potato protein. All diets were pelleted and formulated to meet or exceed nutrient requirements for growing broiler chickens (CVB, 2018). Titanium dioxide (TiO_2_; 4 g/kg) was added to the diets as inert tracer to measure EPL and protein digestibility.

### Oral isotope administration and sampling procedures

Before ^15^N-isotope administration, at 25 d of age, 6 randomly selected birds per pen were removed for dissection to measure apparent nutrient digestibility and digesta retention time, as reported elsewhere (Dorado-Montenegro et al., 2024). The procedures of ^15^N-isotope solution administration were adapted from Dänicke et al. (2000) as described by (Habibi et al., 2026). The proportion of ^15^N relative to the total nitrogen present in the isotope solution and samples is expressed as atom percentage (atom %). In brief, between d 26 and 31, three birds per pen received daily an oral dose of a ^15^N-isotope solution containing 1.2 g ^15^N-enriched milk protein concentrate (atom % = 2.27%) and 0.12 g ^15^NH_4_Cl (atom % ≥ 98%), with an initial priming dose of milk protein concentrate of 2.4 g at d 26.

Representative spot samples of excreta were collected daily at 08:00 and 13:00 from the d of the last isotope dose (d 31) until 37 d of age. At 37 d of age, all three birds designated for ^15^N-isotope infusion were individually weighed and dissected by injection of 0.5 mL of sodium pentobarbital (500 mg/mL) at the back edge of the skull. Ileal digesta were gently flushed out with saline water and collected in the container. All samples were immediately stored at −20°C until further processing.

### Sample preparation and analytical methods

Prior to analyses, excreta and ileal digesta were pooled per pen, freeze-dried, and ground using a rotor mill (Retsch ZM200, Haan, Germany) at 12000 rpm with a 1 mm screen. All chemical and physical analyses were performed in duplicate.

Uric acid was extracted from excreta following the procedure of Baker (1946) and subsequently quantified using a commercial test kit (Uric Acid liquicolor plus, 10694, Human GmbH, Wiesbaden, Germany). The precipitate remaining after uric acid extraction was collected and considered feces. Feed, ileal digesta, and excreta samples were analyzed for titanium (van Bussel et al., 2010), DM (ISO 6496, 1999) and nitrogen (ISO 16634, 2008). ^15^N-enrichment in feed, digesta, excreta, and feces was analyzed by combustion-isotope ratio mass spectrometry using a Delta C continuous-flow isotope ratio mass spectrometer (Finnigan MAT, Bremen, Germany).

### Water-binding capacity and extract viscosity

Water-binding capacity of diets was analyzed with the following modifications of the method of Jacobs et al. (2015). Diets and deionized water were mixed in a conical tube (50 mL) using a vortex (2,500 × g) for 5 s to ensure thorough mixing the components. Values were reported as g water/g dry sample. Extract viscosity was analyzed as the viscosity of the buffer-soluble fraction of diets after extraction of the aqueous fraction, following the modifications of the method of Dorado-Montenegro et al. (2025). All analyses were performed in duplicate.

### Particle size distribution

Particle size distributions of C-SBH, F-SBH, and F-SBP ingredients were analyzed by dry sieving method (ASABE, 2008). Particle size distributions of C-SBH, F-SBH, and F-SBP diets were analyzed by wet sieving method (Wolf et al., 2010). The geometric mean diameter (**GMD**), and geometric standard deviation (**GSD**) of samples were calculated as described by Lyu et al. (2020).

### Calculations

^15^N-enrichmen above background enrichment (expressed as atom percentage excess (**APE**)) was calculated from ^15^N-enrichment in samples minus the background ^15^N-enrichment as measured in the feed (Table 1). The ^15^N-enrichment in excess of background enrichment in those samples were used to calculate total EPL proportion as follows (Dänicke et al., 2000):

$$EPL\;proportion=\frac{APE_{Sample}}{APE_{Urine}}$$

where APE_Sample_ is the ^15^N-enrichments in excess of background enrichment in ileal digesta or feces. ^15^N-enrichments in excess of background enrichment in urine (APE_Urine_) was used to represent the ^15^N-enrichment in endogenous nitrogen pool, which was extensively discussed in a previous study (Habibi et al., 2026) and was calculated as follows (Dänicke et al., 2000):

$$APE_{Urine}=\frac{\left(APE_{Excreta}\times \;N_{Excreta}\right)-\left(APE_{Feces}\times \;N_{Feces}\right)}{N_{Uric\;acid}\;\times \;1.2}$$

where APE_Excreta_ and APE_Feces_ are the ^15^N-enrichment in excess of background enrichment in excreta and feces. Nitrogen content in feces (N_Feces_; g/kg DM) was calculated as the contribution of feces in excreta:

$$N_{Feces}\;=\;N_{Excreta}\;-\left(N_{Uric\;acid}\;\times \;1.2\right)$$

where N_Excreta_, and N_Uric acid_ are the nitrogen contents (g/kg DM) in excreta and uric acid. The correction factor of 1.2 was used to account for urinary nitrogen contents others than uric acid, including ammonia, urea, and amino acids (Terpstra and de Hart, 1973; Dänicke et al., 2000). Subsequently, total EPL (g/kg DM intake) in ileal digesta and feces were calculated by correcting nitrogen flows (N_Flows_, g/kg DM intake) in ileal digesta and feces for EPL proportion as previously calculated (Deglaire et al., 2009):

$$N_{Flows}\;=\;N_{Sample}\times \left(\frac{T_{Diet}}{T_{Sample}}\right)$$

$$EPL\;=\;N_{Flows}\;\times EPL\;proportion$$

where N_Sample_ is the nitrogen contents (g/kg DM) in ileal digesta or feces and T_Diet_ and T_Sample_ are the concentrations of tracers (Ti) (g/kg DM) in the diet and samples of ileal digesta and excreta. Finally, the apparent ileal digestibility (**AID**) and apparent total tract digestibility (**ATTD**) of protein (%) were corrected for EPL to quantify true ileal digestibility (**TID**) and true total tract digestibility (**TTTD**) of protein (%).

$$AID\;or\;ATTD\;of\;protein\;=\left(\frac{N_{Diet}-\;N_{flows}}{N_{Diet}}\right)\times \;100\%$$

$$TID\;or\;TTTD\;of\;protein\;=\left(\frac{N_{Diet}-\left(N_{flows}-\;EPL\right)}{N_{Diet}}\right)\times \;100\%$$

where N_Diet_ is the concentration of nitrogen (g/kg DM) in diet.

**Table 1:** Ingredient and nutrient composition of experimental diets (in g/kg as-fed, unless indicated otherwise) fed to female broilers between 10 and 37 days of age.

Item	C-SBH / F-SBH^1^	C-SBH-AX / F-SBH-AX^2^	F-SBP^3^
Ingredient composition			
Maize	450.0	450.0	450.0
Maize starch	175.1	126.1	176.1
Soybean hulls	120.1	120.1	-
Arabinoxylans	-	50.0	-
Sugar beet pulp	-	-	150.0
Soy protein concentrate	126.0	126.0	126.0
Sugar	28.4	28.4	-
Potato protein	1.5	1.5	-
Fish meal	24.8	24.8	24.8
Soybean oil	15.0	15.0	15.0
Potassium carbonate	3.9	3.9	3.9
Sodium bicarbonate	2.0	2.0	2.0
L-Lysine	5.0	5.0	5.0
D-Methionine	4.1	4.1	4.1
L-Threonine	2.7	2.7	2.7
L-Tryptophane	0.2	0.2	0.2
L-Isoleucine	1.2	1.2	1.2
L-Arginine	3.2	3.2	3.2
L-Leucine	0.5	0.5	0.5
L-Valine	2.4	2.4	2.4
Mineral and vitamin premix^4^	5.0	5.0	5.0
Monocalcium phosphate	11.7	11.7	11.7
Salt	1.2	1.2	1.2
Calcium carbonate	9.0	8.0	8.0
Cobalt-EDTA	1.0	1.0	1.0
Titanium dioxide	4.0	4.0	4.0
Polyethylene glycol	2.0	2.0	2.0

Calculated nutrient composition^5^			
GE^6^ (MJ/kg)	17.6	-	17.4
AME^7^ (MJ/kg)	12.7	-	13.2
Dig lys^8^	11.1	-	10.8
Dig met + cys^8^	7.2	-	7.1
Dig thr^8^	7.6	-	7.3
Ca	7.2	-	7.5
Available P	2.9	-	3.0

Analyzed nutrient composition			
DM	902.1	900.7	902.2
CP^9^	171.8	180.7	168.7
Fat	35.5	37.7	33.6
Ash	50.6	51.4	55.5
Total non-starch polysaccharides	116.5	139.6	109.0
Background ^15^N-enrichment	0.3666	0.3666	0.3665

1 C-SBH: coarse soybean hulls (intact, GMD±GSD = 543±128 μm) and F-SBH: fine soybean hulls (ground by a hammer mill using a 1 mm screen, GMD±GSD = 532±125 μm).

2 Soluble wheat arabinoxylans (AX) (NAXus®, BioActor BV, Maastricht, The Netherlands).

3 F-SBP: fine sugar beet pulp (ground by a hammer mill using a 4 mm and subsequently a 1 mm screen, GMD±GSD = 552±141 μm).

4 Premix provided per kilogram of diet: Vitamin A (retinyl acetate), 10,000 IU; Vitamin D_3_ (cholecalciferol), 2.500 IU; Vitamin E (dl-a-tocopherol), 50 mg; Vitamin K_3_ (menadione), 1.5 mg; Vitamin B_1_ (thiamin), 2.0 mg; Vitamin B_2_ (riboflavin), 7.5 mg; Vitamin B_6_ (pyridoxin-HCl), 3.5 mg; Vitamin B_12_ (cyanocobalamin), 20 µg; Niacin, 35 mg; D-pantothenic acid, 12 mg; Choline chloride, 460 mg; Folic acid, 1.0 mg; Biotin, 0.2 mg; Iron, 80 mg, as FeSo_4_; Copper, 12 mg, as CuSO_4_; Manganese, 85 mg, as MnO; Zinc, 60 mg, as ZnSO_4_; Iodate, 0.8 mg, as KJ; Selenium, 0.15 mg, as Na_2_SeO_3_.

5 Calculated using data from (CVB, 2018).

6 Gross energy.

7 Apparent metabolizable energy for broilers.

8 Standardized ileal digestible amino acids for broilers.

9 Crude protein (CP) was calculated from analyzed Nitrogen × 6.25.

### Statistical analyses

Observations at pen level (3 birds/pen) were considered the experimental unit for all statistical analyses. Effects of PS of SBH and addition of AX on daily FI, BW, EPL, and digestibility of protein were analyzed using a two-way ANOVA (PROC GLM, SAS, version 9.4, SAS Institute Inc., Cary, NC) with PS (coarse vs. fine) of SBH, AX inclusion (0 vs. 50 g/kg), and their interaction as fixed effects. Differences among means were tested using type III least squares, with Tukey adjustments for multiple comparisons. Substitution of F-SBP for F-SBH was tested using an independent t-test. Model assumptions for both analyses were visually verified using histograms, quantile-quantile plots, and studentized residuals. Homogeneity of variances was assessed using Levene’s test. Data are presented as estimated means and pooled SEM, unless indicated otherwise. Differences were considered significant at *P* < 0.05.

## Results

Grinding reduced GMD of SBH by ∼60% (C-SBH 1134±144 μm; F-SBH 459±85 μm, Figure 1, Supplementary materials).GMD of F-SBP was 465±116 μm. Contrasts in particle size of the resulting pelleted diets were, however, smaller (C-SBH diet 543±128 μm vs. F-SBH diet 532±125 μm vs. F-SBP diet 552±141 μm). The addition of AX to SBH diets resulted in a greater extract viscosity (+19%) and replacing F-SBH with F-SBP resulted in a greater WBC (+18%) (Table 2).

**Table 2:** Physicochemical characteristics of experimental diets fed to female broilers between 10 and 37 d of age^1^.

	Diets^2^				
	C-SBH	F-SBH	C-SBH-AX	F-SBH-AX	F-SBP
Water-binding capacity (g/g)	1.6 ± 0.03	1.8 ± 0.04	1.5 ± 0.06	1.5 ± 0.00	2.2 ± 0.00
Extract viscosity (mPa.s)	1.3 ± 0.03	1.3 ± 0.01	1.6 ± 0.05	1.6 ± 0.08	1.3 ± 0.00

1 Data are presented as mean and standard deviation of duplicate analyses.

2 C-SBH = coarse soybean hulls (intact, GMD±GSD = 543±128 μm), F-SBH = fine soybean hulls (ground in hammer mill using a 1 mm screen, GMD±GSD = 532±125 μm), C-SBH-AX = coarse soybean hulls with additional 50 g/kg arabinoxylans, F-SBH-AX = fine soybean hulls with additional 50 g/kg arabinoxylans, F-SBP = fine sugar beet pulps (ground in hammer mill using 4 mm and 1 mm screen, GMD±GSD = 552±141 μm).

Feed intake, as measured between d 22 and 25, did not differ between SBH diets (C-SBH 96±4.2 g/bird/d, F-SBH 97±3.6 g/bird/d, C-SBH-AX 96±6.9 g/bird/d, F-SBH-AX 96±5.6 g/bird/d), but was slightly lower for F-SBP (89±3.8 g/bird/d). Slight variation in final BW was observed between C—OH (2058±153.0 g) and F-OH (2008±217.3 g) diets, but slightly lower than diets containing purified AX (C-SBH-AX 2234±155.9 g, F-SBH-AX 2142±269.1 g) and larger than F-SBP diet (1929±185.9 g).

The addition of AX to SBH diets increased EPL in feces (+0.26–0.43 g/kg DM intake, *P* = 0.020) but not consistently in ileal digesta (+0.01–0.23 g/kg DM intake, *P* = 0.17, Table 3). True ileal protein digestibility was lower in birds fed AX diets compared with SBH diets without AX supplementation (*P* < 0.001), which seemed to be more pronounced for the F-SBH diets (−3.5%-units) compared with C-SBH diets (−1.4%-units) as indicated by the tendency for a PS × AX interaction (*P* = 0.077). True total tract protein digestibility was not affected by addition of AX (*P* = 0.194).

**Table 3:** Total endogenous protein losses (g/kg DM intake) and apparent and true digestibility of protein (%) in female broilers fed diets varying in fiber source (soybean hulls (SBH) vs. sugar beet pulp (SBP)), particle size of SBH, and addition of arabinoxylans (AX), measured at 37 d of age.

Dietary treatment^1^	n^2^	Total endogenous protein losses		Ileal digestibility of protein		Total tract digestibility of protein	
		Ileal digesta	Feces	Apparent	True	Apparent	True
Coarse SBH	8	0.65	1.26	76.2^a^	78.4	78.9	83.1
Fine SBH	8	0.68	1.25	76.4^a^	78.6	80.3	84.4
Coarse SBH + AX	8	0.66	1.69	74.9^a^	77.0	78.4	83.5
Fine SBH + AX	8	0.91	1.51	72.2^b^	75.1	77.4	82.2
Pooled SEM		0.084	0.140	0.62	0.58	0.69	0.69

*P*-values^3^							
Particle size (PS)		0.105	0.537	0.045	0.165	0.783	0.955
Arabinoxylans (AX)		0.167	0.020	< 0.001	<0.001	0.016	0.194
PS × AX		0.220	0.538	0.024	0.077	0.093	0.063
Fine SBH	8	0.68	1.25	76.4	78.6	80.3	84.4
Fine SBP	8	0.99	2.17	75.7	79.0	79.2	86.5
Pooled SEM		0.162	0.474	1.00	0.91	2.07	1.42
*P*-values^4^		0.002	0.004	0.474	0.698	0.307	0.173

1 C-SBH = coarse soybean hulls (intact, GMD±GSD = 543±128 μm), F-SBH = fine soybean hulls (ground in hammer mill using a 1 mm screen, GMD±GSD = 532±125 μm), C-SBH-AX = coarse soybean hulls with additional 50 g/kg arabinoxylans, F-SBH-AX = fine soybean hulls with additional 50 g/kg arabinoxylans, F-SBP = fine sugar beet pulps (ground in hammer mill using 4 mm and 1 mm screen, GMD±GSD = 552±141 μm).

2 Number of replicate pens (3 birds/pen). One replicate of F-SBP treatment has one missing subsample.

3 Model established P-values comparing SBH diets (fixed effects of soybean hulls particle size, arabinoxylans, or their interaction (PS × AX)). In case of significant PS × AX interactions, means within a column lacking a common superscript differ (*P* < 0.05).

4 Model established P-values comparing fine F-SBH and F-SBP diets as fixed effects.

Particle size of SBH did not affect EPL in either ileal digesta nor feces, and did not affect true protein digestibility (*P* > 0.1), other than the above mentioned tendency for PS × AX interaction.

The combined effects of diet properties on EPL, on the one hand, and true digestibility on the other, resulted in lower AID in birds fed AX diets compared with SBH diets without AX supplementation (−1.3–4.2%-units), particularly when fed the F-SBH diets (PS *P* = 0.045, AX *P* < 0.001, PS × AX *P* = 0.024). Similar, but less pronounced, patterns were found for ATTD of protein being 0.5–2.9%-units lower in birds fed AX diets compared with SBH diets without AX supplementation (*P* = 0.016). The substitution of F-SBH with F-SBP increased EPL in ileal digesta (+0.31 g/kg DM intake, *P* = 0.002) and feces (+0.92 g/kg DM intake, *P* = 0.004), but did not significantly influence apparent and true ileal and total tract digestibility of protein.

## Discussion

The aim of this study was to investigate how DF sources varying in physicochemical properties, including PS, solubility, and hydration properties, influence EPL and protein digestibility in broilers. To address this, purified wheat AX, a viscous sDF source, was added to maize-based diets containing either C-SBH or F-SBH as the main iDF source. This design enabled evaluation of the effects of adding viscous sDF to iDF differing in PS. A fifth diet was added in which F-SBH were replaced with F-SBP, a DF source with noticeable hydration properties, allowing to compare intrinsic properties between DF sources. We hypothesized that the presence of AX or replacing SBH with SBP would result in (1) increased EPL and (2) reduced true protein digestibility, whereas particle size reduction of SBH was expected to decrease EPL and true protein digestibility. More importantly, it was expected that the adverse effect of AX on EPL and true protein digestibility may depend on PS of the dietary matrix, and thus on PS of SBH.

### Role of dietary fibers sources in modulating endogenous protein losses

The addition of AX to SBH diets increased EPL, whereas marginal effects of PS of SBH were found. Endogenous protein losses in ileal digesta observed in the present study were lower than basal or total EPL values reported in previous studies (Ravindran et al., 2004; Ravindran and Hendriks, 2004; Danicke et al., 2000). However, the observed differences between dietary treatments in our study are in line with previous studies that have consistently shown that viscous sDF and/or fibers with noticeable hydration properties increase EPL (Leterme et al., 1996, 1998a; Souffrant, 2001). In the current study, supplementation of 50 g/kg AX numerically increased ileal EPL, and diets containing F-SBP resulted in higher EPL than those containing F-SBH, which aligns with these earlier findings.

Arabinoxylans consist of a xylose backbone with arabinose side chains, enabling the formation of a viscous gel in the GIT (Bedford and Classen, 1992; Shelat et al., 2015; Mendez-Encinas et al., 2018; Tiwari et al., 2019). Increased digesta viscosity has been shown to stimulate epithelial cell proliferation in the small intestine (Gee et al., 1996; Wanders et al., 2013, 2014) and to entrap digestive secretions such as bile acids and pancreatic enzymes, thereby limiting their hydrolysis and reabsorption before leaving the small intestine (Souffrant, 2001; Palafox-Carlos et al., 2011; Adeola et al., 2016; reviewed by Jha and Mishra, 2021). These responses could contribute to elevated EPL.

In contrast, EPL associated with SBP may arise primarily from its fermentable fiber fraction. SBP contains higher levels of soluble, highly fermentable components - mainly pectins - compared with SBH (Ivarsson et al., 2014; Feng et al., 2023). Fermentable sDF can stimulate microbial proliferation in the small intestine and hindgut (Ma et al., 2002; Schiavon et al., 2004; Pirman et al., 2007; Lynch et al., 2008; Tan et al., 2016). Indeed, SBP inclusion in pig diets has been associated with increased goblet cell numbers and enhanced mucin production throughout the GIT (Leterme et al., 1998b; Laitat and Philippe, 2015), which may further contribute to elevated EPL.

### Contrasting effects of arabinoxylans and sugar beet pulp on true protein digestibility

Although both AX and SBP increased EPL, only AX reduced true ileal protein digestibility, whereas replacing F-SBH with F-SBP had no significant effect. Because the DF sources used in this study contributed negligible amounts of protein, differences in true protein digestibility are likely attributable to interactions between DF physicochemical properties and other dietary components. Formation of viscous gels promoted by the presence of AX may hinder interactions between digestive enzymes and nutrients, reduce nutrient diffusion to the epithelial surface, and ultimately impair nutrient absorption (Maisonnier et al., 2001; Silva et al., 2013; Sadeghi et al., 2015; Lentle and de Loubens, 2015; Henn and Alim, 2025). In contrast, SBP did not influence true protein digestibility, despite this DF source increased EPL, as also reported previously for pigs (Lynch et al., 2008). Although SBP has notable hydration capacity, its relatively low gelling ability (Phatak et al., 1988) may explain the lack of effects on true protein digestibility. Previous studies reported that apparent digestibility of CP between SBP and oat hulls did not differ (González-Alvarado et al., 2010; Jiménez-Moreno et al., 2013), despite SBP displaying greater water-holding and swelling capacity compared with oat hulls (Jiménez-Moreno et al., 2013).

### Impact of particle size of soybean hulls on digestion processes

Although coarse SBH were expected to enhance gizzard contractions and prolong gastric digesta retention time, thereby increasing EPL through stimulation of digestive secretions and physical abrasion of the gastrointestinal mucosa, only marginal differences were observed between diets containing coarse and fine SBH. Relative gizzard weight, retention time of solid and liquid digesta in the proventriculus and gizzard, and apparent nutrient digestibility were not affected by SBH particle size, as previously reported in a paper of the same study (Dorado-Montenegro et al., 2024).

Although grinding reduced GMD of SBH by ∼60% and considerably reduced the fraction of coarse particles ≥ 0.63 mm (C-SBH ∼60% vs. F-SBH ∼23%, w/w), the contrast in PS at diet level was much smaller (Figure 1, Supplementary materials). It may be postulated the difference in PS between the diets was insufficient to markedly influence digestion processes. Remarkably, the effect of AX on AID of protein was found to depend on PS of SBH; where AX reduced AID of protein only when added to the F-SBH diet but not for the C-SBH. Similar patterns, but less pronounced were observed for TID and ATTD of protein, as indicated by the tendencies for PS × AX interactions (*P* < 0.1). Speculatively, subtle differences in PS and morphometry differently modulated digesta viscosity in C-SBH vs. F-SBH diets. Studies on particle suspensions have demonstrated that the viscosity of suspensions is very sensitive to the size of particles present (Senapati et al., 2010; Konijn et al., 2014). These findings indicate that although PS of SBH had a minor effect on protein digestibility, PS of the dietary matrix may modulate the impact of viscous DF on digestion processes, and that coarse particles can partially counteract the adverse effects of AX on protein digestibility.

### Limitations of apparent digestibility in understanding dietary fiber effects

Commonly used digestibility assays to assess the bioavailability of proteins and amino acids in poultry feed ingredients primarily rely - largely for methodological convenience - on measurement of apparent digestibility i.e. the net disappearance of ingested nitrogen or amino acids along the gastrointestinal tract (Mateos et al., 2012; reviewed by Jha and Mishra, 2021; reviewed by Röhe and Zentek, 2021; Vivares et al., 2025). The diverse physicochemical properties of DF ingredients, such as solubility, viscosity, and hydration capacity, can affect apparent digestibility via distinct mechanisms by altering EPL and/or true protein digestibility (Souffrant, 2001; Dégen et al., 2007). Because apparent nutrient digestibility reflects the combined effects of EPL and true digestibility, it provides limited insights into the underlying mode of action and may even mask counteracting DF effects. This was evident in the present study, where interpretation of DF effects based solely on apparent digestibility was clearly compromised. Viscous soluble AX increased EPL and reduced true protein digestibility, resulting in lower apparent protein digestibility, whereas SBP, with high water-binding capacity, increased EPL without affecting true protein digestibility, but also without causing significant reductions in apparent digestibility. Similar limitations of relying solely on apparent digestibility have been reported, for example, in a study with dogs, where apparent total tract digestibility of protein differed between animal- and plant-based diets, while true total tract digestibility did not (Cargo-Froom et al., 2019). Collectively, these observations highlight that apparent protein digestibility does not clearly explain the dietary effects on EPL and/or true protein digestibility, particularly when DF-rich ingredients are included.

In conclusion, our findings confirm that DF can exert heterogenous effects on EPL, as well as on nutrient hydrolysis and absorption (i.e. true protein digestibility) in female broilers. The addition of AX to SBH containing diets increased EPL and reduced true protein digestibility. Particle size of SBH marginally affected digestion, but the interaction between PS and AX on ileal digestibility of proteins indicates that the presence of viscous soluble DF depended on PS of the dietary matrix and that, speculatively, coarse particles can partially mitigate the adverse impact of AX on protein digestibility. Substituting fine SBP for fine SBH, elicited EPL in ileal digesta and feces, but did not influence true protein digestibility. Apparent digestibility was only affected by the addition of AX, but not when SBP was substituted for SBH. This illustrates that apparent protein digestibility does not fully capture DF-induced changes in digestion processes. Taken together, these findings highlight the need for further mechanistic studies to elucidate how dietary fibers with different physicochemical properties influence EPL and nutrient hydrolysis and -absorption, enabling better interpretation of observations at the apparent digestibility level, particularly in the context of the increasing use of fiber-rich by-products as feed ingredients for broiler diets.

### Declaration of AI and AI-assisted technologies in the writing process

During the preparation of this work the author(s) used ChatGPT/Open AI in order to improve readability of the text and language. After using this tool/service, the author(s) reviewed and edited the content as needed and take(s) full responsibility for the content of the publication

## CRediT authorship contribution statement

**Mochammad F. Habibi:** Writing – review & editing, Writing – original draft, Visualization, Methodology, Investigation, Formal analysis, Data curation, Conceptualization. **Sebastian Dorado-Montenegro:** Writing – review & editing, Investigation. **Ana Isabel García-Ruiz:** Writing – review & editing, Funding acquisition, Conceptualization. **Walter J.J. Gerrits:** Writing – review & editing, Funding acquisition, Conceptualization. **Despoina Georgaki:** Writing – review & editing, Investigation. **René P. Kwakkel:** Writing – review & editing, Supervision, Methodology, Conceptualization. **Sonja de Vries:** Writing – review & editing, Validation, Supervision, Project administration, Investigation, Funding acquisition, Conceptualization.

## Disclosures

This study was performed within the framework of the research program Innovational Research Incentives Scheme Veni with project number 15948, which is financed by the Netherlands Organisation for Scientific Research (NWO), Trouw Nutrition, and Wageningen University & Research. The authors declare that there are no conflicts of interest related to the research presented in this manuscript. Additionally, one of the co-authors, Ana Isabel Garcia-Ruiz is the employee of Trouw Nutrition.

## Appendix Supplementary materials

Image, application 1

## Footnotes

Supplementary material associated with this article can be found, in the online version, at doi:10.1016/j.psj.2026.106902.
