# Supplementary material for: Effects of dietary fibers varying in physicochemical properties on total endogenous protein losses and protein digestibility in broilers
Source: Poult Sci. 2026 Apr 7;105(7):106902. doi: 10.1016/j.psj.2026.106902 (PMC13092584; doi:10.1016/j.psj.2026.106902)
Supplement: Supplementary file 1 [file mmc1.docx]

**SUPPLEMNTARY MATERIALS**

**A**

GMD±GSD of coarse SBH = 1134±144 μm

GMD±GSD of fine SBH = 459±85 μm

GMD±GSD of fine SBP = 465±116 μm

**B**

GMD±GSD of coarse SBH diet = 543±128 μm

GMD±GSD of fine SBH diet = 532±125 μm

GMD±GSD of fine SBP diet = 552±141 μm

Figure 1. (A) Weight fraction of coarse or fine soybean hulls (SBH), or fine sugar beet pulp (SBP) recovered at sieves with various mash sizes during dry sieving analyses. (B) Weight fraction of coarse or fine SBH, or fine SBP diets recovered at sieves with various mash sizes during wet sieving analyses. Bars represent mean of two replicate measurements. Bars represent mean of two replicate measurements. Error bars represent SEM. GMD: geometric mean diameter, GSD: geometric standard deviation.
